# Empirically-supported and non-empirically supported therapies for bulimia nervosa: retrospective patient ratings

**DOI:** 10.1186/2050-2974-1-41

**Published:** 2013-11-04

**Authors:** Lucy Serpell, Blake Stobie, Christopher G Fairburn, Rachel van Schaick

**Affiliations:** 1 Research Department of Clinical, Educational and Health Psychology, University College London, Gower Street, London WC1E 6BT, UK; 2 Eating Disorders Service, North East London NHS Foundation Trust, Porters Avenue Health Centre, 234 Porters Avenue, Dagenham, Essex RM8 2EQ, UK; 3 National Institute for Health Research (NIHR) Biomedical Research Centre for Mental Health at South London and Maudsley NHS Foundation Trust and Institute of Psychiatry, King’s College London, Denmark Hill, London SE5 8AF, UK; 4 Department of Psychiatry, University of Oxford, Warneford Hospital, Oxford OX3 7JX, UK; 5 Present address: Looked After Children’s Team, Children and Young People’s Services, London Borough of Hackney, 1 Hillman Street, London E8 1DY, UK

**Keywords:** Bulimia nervosa, Eating disorders, Cognitive behaviour therapy, CBT, Evidence based treatment, Outcome

## Abstract

**Background:**

Empirically supported therapies for bulimia nervosa include cognitive behaviour therapy and interpersonal therapy. Whilst these treatments have been shown to be effective in multiple randomised controlled trials, little research has investigated how they are perceived by patients who receive them. This study investigated whether empirically-supported psychological therapies (ESTs) are associated with superior self-rated treatment outcomes in clients with Bulimia Nervosa (BN).

**Results:**

98 adults who had received psychological therapy for BN in the United Kingdom completed a questionnaire which retrospectively assessed the specific contents of their psychological therapy and self-rated treatment outcomes.

Around half the sample, fifty three participants reported receiving an EST. Fifty of these received Cognitive Behaviour Therapy (CBT) and three Interpersonal Therapy (IPT). Where therapy met expert criteria for Cognitive Behaviour Therapy for Bulimia Nervosa (CBT-BN, an EST) participants reported superior treatment outcomes than those who appeared to receive non-specialist cognitive-behavioural therapy. However, self-rated treatment outcomes were similar overall between those whose therapy met criteria for ESTs and those whose therapy did not.

**Conclusions:**

The findings offer tentative support for the perceived helpfulness of CBT-BN as evaluated in controlled research trials. Cognitive-behavioural therapies for BN, as they are delivered in the UK, may not necessarily be perceived as more beneficial by clients with BN than psychological therapies which currently have less empirical support.

## Background

Treatment guidelines for the United Kingdom recommend that individuals with BN should be offered 16-20 sessions of a specialist form of Cognitive Behaviour Therapy (CBT) called CBT-BN. If clients do not want or do not respond to CBT-BN, Interpersonal Psychotherapy (IPT) should be offered. For clients with Eating Disorder Not Otherwise Specified (EDNOS), the specified approach for the most similar eating disorder should be followed [1]. Similar treatment guidelines exist in the US [2].

Despite this guidance, a large proportion of sufferers of BN are not receiving the recommended treatment [3-5]. Studies surveying clinicians have found that they tend to apply a range of psychodynamic and cognitive-behavioural interventions to work with people with eating disorders [6,7]. Only a minority of clinicians use CBT as their primary approach to eating disorders and fewer than 4% of general practitioners use national guidelines to inform their treatment decisions [8]. The lack of availability of IPT is more pronounced than that of CBT. Currently there are only six centres for professional IPT training in the UK, compared to numerous CBT training centres [9].

A further concern regarding treatment for people with BN is that some sufferers may be receiving psychological therapy that is labelled as CBT but does not include the core components of the treatment which have been evaluated in research trials. In the treatment of anxiety disorders, similar ‘off-model’ CBT is associated with poorer treatment outcomes [10,11].

The majority of research investigating treatment for BN has utilised outcome measures which measure eating disorder symptom levels but has neglected to evaluate clients’ perspectives on the helpfulness of treatments. Users of mental health services commonly complain that they are given inadequate information and excluded from treatment decisions [12]. In response to this, there has been an increasing focus on taking account of the views of clients in developing and evaluating treatments [13,14]. It is important to investigate the views of clients so that they can be combined with research evidence and clinical expertise, in order to develop treatments that are effective and acceptable to clients [15]. This is arguably particularly important in the field of eating disorders where clients are often ambivalent about receiving treatment [16-19]. No research to date has analysed the specific components of psychological therapy and related self-rated treatment outcomes in clients with BN.

This study aimed to investigate whether receiving ESTs, as they have been evaluated in randomised controlled trials (RCTs), is associated with superior treatment outcomes from the clients’ perspective compared to psychological therapies which currently have less empirical support. It was expected that, given the lack of training opportunities for IPT in the UK, only a minority of patients would report receiving this approach. It was hypothesised that participants who recalled having received ESTs for BN (CBT or IPT), particularly CBT-BN, would report greater self-rated treatment gains than those who recalled having received non-ESTs.

## Methods

### Design

An on-line questionnaire was designed to retrospectively assess the specific contents of participants’ most recent set of psychological therapy for BN (The Bulimia Treatment History Questionnaire; BTHQ). The BTHQ also included a measure of symptom severity at the time participants commenced the therapy. Potential participants from across the United Kingdom were made aware of the study through a number of strategies (see Procedure). If interested in the study, potential participants could access an on-line information sheet, consent form and BTHQ.

### Participants

Participants were eighteen or over and recalled having received psychological therapy for BN or EDNOS BN-Subtype. Participants were excluded if they met criteria for Anorexia Nervosa (AN) or EDNOS AN-Subtype at the start of their therapy.

### Procedure

Ethical approval was granted from the local research ethics committee. Potential participants were made aware of the study with the help of the eating disorder charity *Beat* (UK Eating Disorders Association). Awareness of the study was mainly raised through on-line methods, i.e. web-posts and emails which gave a brief description of the study and provided a hyper-link to further information. The specific strategies employed were as follows: a) Posts were displayed on *Beat’s* social networking websites and on the websites of other mental health charities and organisations; b) *Beat* emailed their professional members’ network; c) An email was sent to all staff and students of the researcher’s university; d) A poster advertising the study was displayed in a number of eating disorder treatment centres, general practices and university campuses nationally.

### Measures

#### Contents of therapy and self-rated treatment outcomes

The Bulimia Treatment History Questionnaire (BTHQ, developed for current study) is an adapted form of ‘The OCD Treatment History Questionnaire’ [10]. It includes items addressing demographics, the onset and course of the participants’ eating disorder and the most recent set of psychological therapy which the respondent received for their eating disorder. An early item asks participants to chose from a list of possible therapy types including both empiricially supported therapies (CBT, IPT) and those without current empirical support (e.g. humanistic, cognitive analytic or supportive therapy). Possible therapy types were generated from discussion with patients and clinicians and are shown in Table 1. The content of psychological therapy is then assessed using 36 statements regarding therapy (see Appendix A) to which participants are asked to indicate whether they recall the item being a part of their therapy with ‘yes’/ ‘no’ response options. Items also rate the improvement in their eating disorder symptoms (ED Treatment Gains) and improvement in other aspects of their well-being (General Treatment Gains). Participants rate treatment gains on a scale of 0-100, where 0 is ‘no improvement’ and 100 is ‘total recovery’. The BTHQ also contained questions regarding the eating disorder symptoms which the individual was suffering from, as well as weight and height at the time of commencing therapy. The BTHQ was designed in collaboration with experts in the fields of CBT (BS) and eating disorder treatments (LS and CF). It was piloted by two individuals who had received psychological therapy for an eating disorder and by the last author, a Clinical Psychologist who specialises in the psychological treatment of eating disorders (LS).

**Table 1:** Respondent Demographics and Characteristics of Psychological Therapy

	**n (N = 98)**	**%**
*Gender*		
Female	96	97.96
Male	2	2.04
*Highest Educational Level*		
Degree or Diploma	57	58.16
AS or A-levels	33	33.67
G. C. S. E.s	8	8.16
	n (N = 98)	%
*Occupational Status*		
Employed	54	55.10
Studying	36	36.73
Full time parent/ carer	4	4.08
Sick leave	2	2.04
Unemployed	1	1.02
Retired	1	1.02
*Treatment Provider*		
NHS	78	79.59
Private	18	18.37
Not sure	2	2.04
	n (N = 98)	%
*Format*		
Outpatient	92	93.88
Day-patient	6	6.12
Inpatient	4	4.08
*Professional*		
Psychologist	37	37.76
Counsellor	19	19.39
Psychiatrist	13	13.26
Nurse Therapist	10	10.20
Community Psychiatric Nurse	3	3.06
Psychodynamic Psychotherapist	3	3.06
Family Therapist	2	2.04
Other	9	9.18
	n (N = 98)	%
*Format*		
Not Sure	2	2.04
	n (N = 98)	%
*Most recent type of Psychological Therapy*^*a*^		
Cognitive Behaviour Therapy	50	51.02
Counselling/ Supportive Therapy	11	11.22
Not Sure	11	11.22
Psychodynamic Psychotherapy	4	4.08
Interpersonal Psychotherapy	3	3.06
Eating Disorder Group Therapy	3	3.06
Humanistic Therapy	2	2.04
Dialectical Behaviour Therapy	2	2.04
Cognitive Analytic Therapy	2	2.04
Behaviour Therapy	1	1.02
General Group Therapy	1	1.02
Over-eaters Anonymous 12-step Programme	1	1.02
	n (N = 98)	%
*Most recent type of Psychological Therapy*^*a*^		
Family or Couples Therapy	1	1.02
Other	6	6.12

^a^i.e. Therapy assessed by BTHQ.

### Data analysis

A power calculation informed by prior research [11] indicated that at a power level of 0.8, a sample size of 57 participants would be necessary to detect differences in ED Treatment Gains, and a sample size of 126 would be necessary to detect differences in General Treatment Gains. Unfortunately due to resource constraints only 98 participants were recruited (and 79 used in inferential statistical analysis), meaning the analysis in regard to General Treatment Gains was underpowered.

Data regarding 98 participants was analysed descriptively. Participants who were unsure what type of psychological therapy they had received (N = 11) or had missed more than 25% of their allocated sessions (N = 8) were excluded from statistical analysis. 79 participants were therefore included in inferential statistical analysis (as there was no overlap between participants excluded for the above two reasons).

#### CBT quality

Participants who recalled having received CBT were classified into groups dependent on whether they were judged to have received evidence based psychological therapy. This was defined as therapy delivered in the way it has been evaluated in controlled research trials. Criteria for these classifications were constructed for both CBT and IPT by two expert clinicians / researchers in the field of CBT for eating disorders, CF and LS. However, because of the very small number of participants who reported receiving IPT, only data on CBT is presented in this paper. Criteria for the classification of CBT are shown in Tables 2 and 3.

**Table 2:** Classification of CBT-BN (N = 44)

***Questions Used to Classify CBT-BN***	**Answered ‘Yes’**	
	**n**	**%**
1. ‘We discussed the relationship between binge-eating and dieting.’	29	65.90
2. ‘We talked about any issues I had about looking at my own body (for example, frequently checking parts of my body or avoiding looking at parts of my body).’	27	61.36
3. ‘I was given advice about how to, or was encouraged to, establish a regular pattern of eating.’	38	86.36
4. ‘I was provided with information about weight and eating (for example, the consequences of binge-eating, self-induced vomiting, and laxative abuse).’	29	65.90
5. ‘We discussed how I could stop dieting or how I could stop avoiding eating.’	31	70.45
*Classified as having received CBT-BN*^*a*^	15	34.09
*Classified as having received Standard CBT (i.e.* not *classified as having received CBT-BN)*^*b*^	29	65.91

CBT-BN: Cognitive Behaviour Therapy for Bulimia Nervosa.

CBT: Cognitive Behaviour Therapy.

^a^Answered ‘Yes’ to all questions 1-5.

^b^Answered ‘No’ to any of questions 1-5.

**Table 3:** Classification of Adequate CBT (N = 44)

***Questions Used to Classify Adequate CBT***	**Answered ‘Yes’**	
	**n**	**%**
1. ‘My therapist and I both had an active role in treatment (for example, we planned how to spend therapy sessions and tasks that I would do).’	31	70.45
2. ‘The therapy involved carrying out regular ‘homework’ or self-help tasks outside of the therapy sessions.’	37	84.09
3. ‘I monitored my eating habits in a diary or record.’	32	72.73
4. ‘My therapist explained the treatment approach and the rationale behind it.’	30	68.18
*Classified as having received Adequate CBT*^*a*^	17	38.64
*Classified as having received Inadequate CBT (i.e.* not *classified as having received Adequate CBT)*^*b*^	27	61.36

CBT: Cognitive Behaviour Therapy.

^a^Answered ‘Yes’ to all Questions 1-4.

^b^Answered ‘No’ to any of questions 1-4.

Two categories of CBT quality were constructed: *1. CBT-BN*: In order to be classified as having received CBT-BN, participants must have recalled engaging in CBT and answered ‘yes’ to five specified questions relating to CBT-BN (Table 2). If participants answered ‘no’ to any of these five questions relating to CBT-BN they were classified as having received *Standard CBT*. The classifying items were formulated by consulting the CBT-BN treatment manual [20] to determine key components of the treatment (RVS and LS). Key components were translated into items deemed integral to CBT-BN. The items were then cross-checked by CF, one of the developers of CBT-BN.

*2. ‘Adequate CBT’*: This category was related to core components of CBT. Participants must have recalled engaging in CBT and answered ‘Yes’ to four questions relating to general CBT technique (Table 3). If participants answered ‘no’ to any of these five questions relating to Adequate CBT they were classified as having received *Inadequate CBT*. The items were formulated by RVS and LS using their clinical knowledge and referring to treatment guides. Items were then cross-checked by LS and BS, both experienced CBT therapists.

CBT-BN was classified separately to Adequate CBT in order to try and ascertain specific components of treatment that were perceived as helpful by clients. Criteria for deciding whether adequate IPT had been received by participants were also developed. As only two participants recalled receiving IPT this analysis was discarded.

### Statistical analysis

All statistical analysis was conducted using SPSS version 14.0. Prior to any statistical analysis the data were checked for normality and homogeneity of variance. Due to significant non-normality of outcome variables non-parametric tests (Mann Whitney U) were used. To control for the fact that multiple significance tests were carried out, Bonferroni corrections were applied.

## Results

### Participants and psychological therapy

98 participants with BN completed the BTHQ between July 2010 and January 2011. Demographic data and characteristics of the psychological therapy recalled by participants is summarised in Table 1. The range of time elapsed between start of therapy and completion of the BTHQ was 0 to 16 years. The modal year treatment started was 2009, i.e. 1-2 years previously (21 participants). Three participants reported engaging in the therapy described before 2000, i.e. more than 10 years previously. Ninety two participants (94%) reported beginning the therapy described after January 2004 (when current UK treatment guidance was published).

### Eating disorder symptoms and BMI

Based on symptoms reported, all participants therefore broadly met criteria for BN, this term is used throughout this paper. 18 participants reported being underweight (18.4%: BMI < 18.5), 60 were in the healthy range (61.2%: BMI 18.5 – 24.9), 11 were overweight (11.2%: BMI 25 – 29.9) and 9 were obese (9.2%: BMI 30+) at the time of commencing therapy.

### Type of psychological therapy

Table 1 shows the different types of psychological therapy that participants recalled most recently engaging in. Just over half of participants recalled engaging in the nationally-recommended treatments of CBT (51.02%) or IPT (3.06%).

### Severity of disorder Pre-treatment

Prior to hypothesis testing, Global EDE-Q scores were compared between groups, to investigate whether there were pre-treatment differences in severity of eating disorder. There was found to be no significant differences in severity of eating disorder symptoms (Global EDE-Q scores) between any of the groups compared for self-rated treatment outcomes.

### Self-rated treatment gains

Tables 2 and 3 show the proportions of participants who were deemed as having received the different classifications of CBT quality. Only 34% of participants were classified as having received CBT-BN (Table 2) and only 39% of participants were classified as having received Adequate CBT (Table 3). Table 4 shows mean self-rated treatment gains for participants who recalled having received CBT, according to the different classifications of CBT Quality. Table 4 also shows mean self-rated treatment gains for participants who recalled receiving either CBT or IPT (EST group) and participants who recalled receiving a treatment not recommended in NICE guidance (Non-EST group).

**Table 4:** Mean Self-Rated Treatment Gains by CBT Quality Classifications

	**CBT-BN**	**Standard CBT**	**Adequate CBT**	**Inadequate CBT**	**ESTs**	**Non-ESTs**
N	15	29	17	27	46	33
Eating Disorder Treatment Gains M (SD)	62.67 (22.82)	30.34 (28.56)	44.12 (32.32)	39.63 (30.09)	41.96 (31.01)	44.91 (32.17)
General Treatment Gains M (SD)	65.67 (22.59)	32.52 (30.34)	51.53 (32.58)	38.96 (31.10)	44.30 (31.72)	49.18 (31.22)

CBT: Cognitive Behaviour Therapy.

CBT-BN: Cognitive Behaviour Therapy for Bulimia Nervosa.

ESTs: Empirically-Supported Psychological Therapies (CBT and IPT).

In regard to improvements in eating disorder symptoms, those who were classified as having received CBT-BN reported the highest self-rated treatment gains (M = 62.67, SD = 22.82). Those who were classified as having received a treatment labelled as CBT which was not judged as containing the core components of CBT-BN (i.e. Standard CBT) reported the lowest self-rated treatment gains (M = 30.34, SD = 28.56).

### CBT-BN

Participants who received CBT-BN reported significantly superior ED Treatment Gains [U(78) = 88.5, z = -3.214, p = .001] and General Treatment Gains [U(78) = 91.0, z = -3.155, p = .002] than participants who recalled having received Standard CBT. No significant differences were found between those who received CBT-BN and those who received therapies which have less current empirical support (Non-ESTs), for either ED Treatment Gains [U(78) = 169.0, z = -1.752, p = .080] or General Treatment Gains [U(78) = 172.5, z = -1.676, p = .094].

### Adequate CBT

There were no significant differences in self-rated treatment gains for participants who had received Adequate CBT and Inadequate CBT, in regard to ED Treatment Gains [U(78) = 212.5, z = -.412, p = .68] or General Treatment Gains [U(78) = 179.5, z = -1.214, p < .78]. There were no significant differences in self-rated treatment gains between participants who had received Adequate CBT and participants who had received psychological therapies which currently have less empirical support (Non-ESTs) in regard to ED Treatment Gains [U(78) = 279.5, z = -.021, p < .99] and General Treatment Gains [U(78) = 266.5, z = -.288, p < .75].

### CBT-BN and adequate CBT

Nine participants (20%) were classified as having received both CBT-BN and Adequate CBT. These participants reported mean ED Treatment Gains of 63.38 (SD = 27.36) and mean General Treatment Gains of 75.0 (SD = 19.04). Statistical analysis was not performed in regard to this group due to the small sample size.

### Treatment histories

Findings relating to the course and treatment of participants' eating disorders are shown in Table 5.

**Table 5:** Participant Eating Disorder Course and Treatment

	**n (N = 98)**	**Range (years)**	**M (SD) (years)**
*Age Symptoms First Developed*	98	6 - 22	13.6 (3.25)
*Age Symptoms Began Interfering Significantly with Life*	98	11 - 43	16.5 (4.32)
*Age Professional Diagnosis*	92	11 - 54	19.2 (6.00)
*Age First Sought Professional Help*	98	11-54	19.8 (6.57)
*Age First Offered Treatment*	96	12-54	20.4 (7.48)
*Age First Received Treatment*	98	12-54	21.2 (7.31)
*Duration Between Seeking Professional Help and Being Offered Treatment*	88	0-18	0.84 (3.80)

## Discussion

This study aimed to investigate whether, for people with BN, psychological therapies with much empirical support are associated with superior self-rated treatment gains than psychological therapies with less current empirical support. The results of the study were mixed in regard to this question.

### CBT-BN

Participants who recalled engaging in therapy classified by researchers as CBT-BN rated their treatment gains more highly on average than participants who had received non-specialist CBT. However, the differences in self-rated treatment gains between Adequate CBT and Inadequate CBT were minimal and were not found to be statistically significant. Those clients who were classified as having received both Adequate CBT and CBT-BN rated their treatment outcomes very similarly to those who were classified as only receiving CBT-BN. Although there are numerous potential threats to internal validity to consider (see below), taken together these findings hint that it may be the core techniques of CBT-BN that are perceived as helpful by clients, rather than generic cognitive-behavioural techniques such as self-monitoring or homework setting. CBT-BN is derived from the cognitive model of BN [21] and hence the items used to classify CBT-BN in this study were based on techniques derived from this model. If confirmed in future studies, the findings could be viewed as evidence to support the utility of the specific cognitive-behavioural model of BN.

It was interesting that no significant differences were found between CBT-BN and Non-ESTs for either ED Treatment Gains or General Treatment Gains. However it is possible that this comparison was underpowered and hence the lack of a significant difference may have been due to a type II error.

The small (20%) overlap between the Adequate CBT group and the CBT-BN group is surprising. It might be expected that most or all of those in the CBT-BN group would have also been rated as receiving adequate CBT. This suggests that further validation of the CBT quality classifications should be the focus of future research.

### ‘Sub-standard CBT?’

The Standard CBT group recalled having received CBT for BN but the recalled contents of their therapy did not meet minimum criteria for CBT-BN, a recommended treatment, as classified by the researchers. Therefore it is arguable that the Standard CBT group may have been receiving a treatment that had been ‘labelled’ as CBT, rather than CBT-BN, which has been found to be efficacious in research trials. As the Standard CBT group perceived their therapy to be less helpful than the CBT-BN group, the results could be interpreted as a tentative warning that although cognitive-behavioural techniques are recommended in UK national guidelines for the treatment of BN, this should not be interpreted simply as a blanket prescription for generic CBT: treatments should include specific components that have been shown to be beneficial in reducing bulimic symptoms.

### ‘Evidence-based’ treatments

Only just over half of individuals who had received a psychological therapy for BN had received a therapy that was recalled as being described as CBT or IPT. Furthermore, of those participants who recalled engaging in a treatment labelled as CBT (n = 44), only 15 (34.1%) were deemed to have received CBT-BN when the recalled contents of the therapy was examined and categorised by the researchers. However, contrary to prediction, individuals who had received the empirically-supported treatments of CBT and IPT rated their treatment gains very similarly, on average, to those who had received a variety of psychological therapies not indicated in national guidance. This surprising result differs from findings of similar studies with sufferers of anxiety disorders [10] and the results of numerous RCTs that have found CBT and IPT to be superior to other psychological treatments for eating disorders [22]. Perhaps the most surprising finding is that although there was a trend for self-rated treatment gains to be higher in the CBT-BN group than the Non-EST group, this difference was not found to be statistically significant, suggesting that even when CBT is delivered ‘at its best’, it is not perceived as significantly more beneficial by clients than other psychological therapies which currently have less empirical support (and are not indicated in UK treatment guidance).

One must be cautious in making interpretations on the basis of this finding as there are a number of variables which were not measured which may have influenced self-rated treatment gains. Levels of co-morbidity of psychological disorders were not measured, and could be hypothesised to play a significant role in self-rated treatment gains (e.g. treatment could be perceived as less effective by someone with a co-morbid personality disorder). Other factors that were not measured and could conceivably have influenced self-rated treatment gains include client ethnicity, duration of treatment and therapist characteristics.

### Treatment histories

A secondary aim of this study was to add to existing evidence regarding self-reported treatment histories of sufferers of BN. In line with previous findings, this study found long delays between symptom onset and seeking treatment and delays between seeking and receiving treatment [12,23]. Eating disorders are often hidden by sufferers [24] and this is likely to account in part for these concerning findings. It is likely that issues relating to the availability of psychological therapy for BN also in part account for such findings.

### Study strengths and limitations

This study is the first, to the authors’ knowledge, to investigate the relationship between the contents of psychological therapy and self-rated treatment outcomes in a sample of individuals with BN. It benefits from investigating clients’ views on treatment, which have been previously under researched in the field of eating disorders. It has been argued that asking clients how much the therapy helped the problem that led them to treatment is a valuable method of measuring clinical significance, as it leaves little doubt regarding the human significance of the treatment [25]. Further strengths of the study include the fact that the sample was drawn from across the whole of the UK, both of which increase the generalisability of the findings.

There are a number of limitations to the current study which mean that findings should be considered preliminary. One key limitation is that the study assessed contents of therapy and perceived treatment gains solely on a retrospective basis and therefore relied on the recall of participants of treatment which may have occurred over 10 years previously. It would be important for future studies to focus on more recent treatment experiences. However, in order to recruit an acceptable sample within the time and funding constraints of the current exploratory study, it was decided to include the small number of participants who were reporting on treatment received many years previously.

The ratings scales for evaluating adequate CBT and CBT-BN have not been used in prior research. Further validation of these scales, ideally in a sample currently undergoing therapy, would be an important focus for furture research. However, the scales were based on previously published scales for the evaluation of CBT on OCD, and were developed with significant input from the lead author on treatment manuals for both CBT and IPT for eating disorders.

The representativeness of the sample is also questionable, because the recruitment methods employed mainly targeted people who were using on-line eating disorder support groups and all participants were self-selected. Thus the sample may have been representative of those with more severe levels of eating disorders and/or that responded less well to psychological therapy. Such a sample would conceivably be more likely to use support groups and be more motivated to share their experiences than those who had positive treatment experiences.

A number of possible biases may have influenced the findings of the study. For example, it is likely that individuals who liked their therapy also rated their outcomes as more positive. Furthermore, it is possible that those participants who continued to use their CBT skills post-therapy had better recall of their therapy experiences. This may have biased memory in favour of CBT. In order to reduce memory bias, participants were asked about their most recent experience of psychological therapy. However, it is acknowledged that some people with eating disorders receive intermittent and varying levels of support during their eating disorder, and it may have been difficult for such individuals to answer the questions regarding a recent discrete therapy experience.

## Conclusions

This study examined self-rated treatment outcomes for clients who met broad diagnostic criteria for BN. The findings support the perceived helpfulness of CBT-BN. Conversely, the findings suggest that different psychological therapies for BN (which have varying degrees of empirical support), as they are delivered in practice in the UK, are perceived to be equally helpful by clients.

The findings regarding the association between CBT-BN and superior self-rated treatment outcomes have important implications regarding therapeutic mechanisms of action and it is important to know whether they are replicable. A prospective investigation in this area with a larger sample size would be beneficial. Qualitative investigations of clients’ perspectives on treatment would help to shed light on what clients find helpful and why.

More research is needed to investigate the finding regarding similar self-rated treatment gains for therapies with much empirical support and therapies with less empirical support. This would help to ascertain whether this finding relates to methodological limitations of this study, an actual efficacy-effectiveness gap in this area, and/or inadequate scientific evaluation of therapies which currently have less empirical support. Finally, self-rated treatment gains in regard to a new, enhanced version of CBT for eating disorders (CBT-E) [26] should be investigated, as this treatment shows promising treatment outcomes in evaluations to date [27,28].

## Endnotes

^a^ Two participants requested a hard-copy version of the questionnaire. Those wishing to complete the hard-copy version were asked to email the researcher to register an interest in taking part in the study. The researcher then posted the relevant documents which they could return in a supplied stamped addressed envelope.

## Appendix A

Items Used To Determine The Contents Of Therapy

1. My therapist and I both had an active role in treatment (for example, we planned together how to spend therapy sessions and tasks that I would do).

2. My therapist explained that the therapy was designed to help me recognise and work on relevant problems in relationships.

3. We focused mainly on my present and my future rather than my past.

4. We looked at links between my thoughts, feelings and behaviours.

5. We discussed the relationship between binge-eating and dieting.

6. My therapist talked about 'deeper' levels of meaning of which I had not always been aware.

7. We talked about any issues I had about looking at my own body (for example, frequently checking parts of my body or avoiding looking at my body).

8. My therapist told me his/her views about how my present feelings and experiences linked to the past.

9. The therapy involved carrying out regular 'homework' or self-help tasks outside of the therapy sessions.

10. We reviewed my home-work or self-help tasks in our therapy sessions.

11. I was given advice about how to, or was encouraged to, establish a regular pattern of eating.

12. We reviewed important relationships from my past in terms of their positive and negative aspects.

13. We explored repetitive patterns in my relationships with others.

14. I was encouraged to weigh myself once a week (no more and no less).

15. We did not make a plan for how my therapy sessions would be spent; I was encouraged to talk and reflect freely about what was on my mind at the time.

16. I kept records or diaries of my thoughts.

17. I explored alternative or more helpful thoughts.

18. I monitored my eating habits in a diary or record.

19. My therapist implied that exploring the past can help to understand the present better.

20. We explored ways to bring about change in difficult relationships with other people.

21. I was provided with information about weight and eating (for example, the consequences of binge-eating, self-induced vomiting, and laxative abuse).

22. We worked mainly on issues to do with my relationships with others, rather than directly addressing eating, weight and shape.

23. My therapist told me his/her thoughts about how I was relating to him/her (for example if I found him/her critical, helpful or judgemental).

24. We designed and carried out experiments to 'test-out' any problematic or unhelpful thoughts I was experiencing.

25. There was a focus on my early childhood experiences.

26. We carried out a review of my past which looked at the history of my eating problem.

27. My therapist explained the treatment approach and the rationale behind it.

28. We explored my expectations about my relationships with others.

29. We linked symptoms of my eating problem to relationship problems.

30. We worked on problem-solving skills.

31. We focused on things that were keeping the problem going rather than things that contributed to the problem developing.

32. We discussed how I felt about my body.

33. We linked symptoms of my eating problem to difficulty coping with recent changes in my life.

34. We talked about how my relationships with others were going, in terms of how intimate they were, how equal they were, or aspects of my relationships that I found satisfying or unsatisfying.

35. We discussed how I could stop dieting or how I could stop avoiding eating.

36. We made a plan for how I would manage once therapy had ended.

## Competing interests

CGF is supported by a Principal Research Fellowship from the Wellcome Trust (046386). CGF is also the lead author of the leading cognitive behavioural treatment for bulimia nervosa/eating disorders. BS is funded by the National Institute for Health Research (NIHR). The views expressed are those of the author(s) and not necessarily those of the NHS, the NIHR or the Department of Health.

## Authors’ contributions

LS developed the initial concept for the study and oversaw the study, analyses and carried out multiple revisions of the manuscript. RVS developed the proposal, assembled study materials and recruited and tested participants. She also conducted the analyses and wrote the first draft of the manuscript. BS advised on the study design, contributed to the therapy rating scales and contributed to drafts of the manuscript. CGF contributed to the development of the therapy rating scales and contributed to drafts of the manuscript. All authors read and approved the final manuscript.
